# Effect of Spinal Cord Compression on Local Vascular Blood Flow and Perfusion Capacity

**DOI:** 10.1371/journal.pone.0108820

**Published:** 2014-09-30

**Authors:** Mohammed Alshareef, Vibhor Krishna, Jahid Ferdous, Ahmed Alshareef, Mark Kindy, Vijaya B. Kolachalama, Tarek Shazly

**Affiliations:** 1 College of Medicine, Medical University of South Carolina, Charleston, SC, United States of America; 2 Department of Neurosciences, Medical University of South Carolina, Charleston, SC, United States of America; 3 Biomedical Engineering Program, University of South Carolina, Columbia, SC, United States of America; 4 Department of Biomedical Engineering, Duke University, Durham, NC, United States of America; 5 Ralph H. Johnson VA Medical Center, Charleston, SC, United States of America; 6 Charles Stark Draper Laboratory, Cambridge, MA, United States of America; 7 Department of Mechanical Engineering, University of South Carolina, Columbia, SC, United States of America; University of Toronto, Canada

## Abstract

Spinal cord injury (SCI) can induce prolonged spinal cord compression that may result in a reduction of local tissue perfusion, progressive ischemia, and potentially irreversible tissue necrosis. Due to the combination of risk factors and the varied presentation of symptoms, the appropriate method and time course for clinical intervention following SCI are not always evident. In this study, a three-dimensional finite element fluid-structure interaction model of the cervical spinal cord was developed to examine how traditionally sub-clinical compressive mechanical loads impact spinal arterial blood flow. The spinal cord and surrounding dura mater were modeled as linear elastic, isotropic, and incompressible solids, while blood was modeled as a single-phased, incompressible Newtonian fluid. Simulation results indicate that anterior, posterior, and anteroposterior compressions of the cervical spinal cord have significantly different ischemic potentials, with prediction that the posterior component of loading elevates patient risk due to the concomitant reduction of blood flow in the arterial branches. Conversely, anterior loading compromises flow through the anterior spinal artery but minimally impacts branch flow rates. The findings of this study provide novel insight into how sub-clinical spinal cord compression could give rise to certain disease states, and suggest a need to monitor spinal artery perfusion following even mild compressive loading.

## Introduction

The incidence of spinal cord injury (SCI) in the United States is approximately 12,000 individuals annually, with causes including various forms of trauma and non-traumatic diseases [1]. Spinal cord compression is seen in majority of SCI patients [2], and may involve anterior, posterior, circumferential, anteroposterior, and less frequently lateral compression. Acute anterior compression of the spinal cord can result from disc herniation, fracture dislocation, or anterior epidural mass formation (tumor, abscess, hematoma etc.). Anteroposterior compression is observed in facet dislocation without vertebral dislocation (perched or jumped facets with no injury to anterior or posterior longitudinal ligaments) and posteriorly located epidural masses (tumor, abscess, hematoma etc.).

The pathogenesis of SCI is understood in two distinct but interrelated phases, distinguished as primary and secondary injury [3], [4]. Primary injury refers to tissue damage sustained during or immediately following trauma, which includes acute compression, impact, distraction, laceration, and shearing. The neurological dysfunction observed in primary injury involves both direct neurological injury (neuronal component) along with ischemia resulting from alterations in blood flow (vascular component). Secondary injury is attributed to disruption of the blood-spinal cord barrier, the generation of an inflammatory response, and local biochemical changes, which together compromise tissue health and neurological function over time [3], [5]. Both primary and secondary injury mechanisms contribute to progressive spinal cord ischemia, which can cause necrosis and irreversible tissue damage if perfusion falls below a critical level, or vascular threshold [6]. Thus, ischemia must be treated in a time-sensitive manner, with evidence that rapid restoration of blood flow may improve patient outcomes [7].

Effective treatment of ischemia in any vascular bed is predicated by a mechanistic understanding of the underlying cause. While there are several hypotheses regarding SCI-induced ischemia including vasospasm due to mechanical damage [8], [9], endothelial damage or swelling [10]–[12], and thrombosis [13], the primary determinants are only partially understood in this clinical indication [3]. However, it is established that the severity and duration of compression together determine the manifestation of clinical symptoms. For example, severe compression beyond a critical level of severity or ‘injury threshold’ causes immediate and significant neurological deficit without return of meaningful function [14], [15]. Alternatively, moderate compression (<35% dural occlusion) is initially asymptomatic [6], but when sustained can dramatically worsen clinical outcomes [16]–[18]. While there are no perceptible neurological deficits in this sub-clinical range of compression, there is electrophysiological evidence of axonal dysfunction as measured by somatosensory evoked potentials [16].

Although spinal compression may be designated by a single directional orientation following SCI (anterior, posterior, etc.), patients typically present with multi-directional and thus more complex compression scenarios. Injuries including ligamentous buckling, facet dislocations, fractures, and hypertrophies can cause a combination of posterior, anterior, and lateral cord compression. Conversely, in injuries such as herniated disks, ventral spurs of uncinate processes, limbus fractures, and ossification of posterior longitudinal ligament (OPLL), stenosis characterized by a reduction in the anteroposterior diameter is caused mainly by compression of the anterior face of the cord [19]–[21].

The complexity of objectively characterizing the mode of compression in turn creates difficulties in identifying the appropriate intervention. Furthermore, due to the size of the spinal vasculature and the limited resolution of current perfusion magnetic resonance imaging (MRI), it is difficult to assess local blood flow and perfusion following SCI. Quantifying blood perfusion in the spinal cord has been attempted using multiple techniques. Some of the approaches require laminectomies, including laser Doppler flowmetry [22], dynamic contrast-enhanced Doppler ultrasonography [23], subdural pressure probe measurements [24], photoplethysmography [25], and a hydrogen clearance technique [26]. These techniques are not feasible for clinical use because of inherent invasiveness, and are only utilized intra-operatively. Other modalities have been employed for external imaging, including microCT imaging [27], infrared spectroscopy (NIRS) [28], and MRI [29]. Typically these approaches are used to examine the effects of factors intended to increase blood perfusion following injury, including hypercapnia, beta2-agonsists, and FGF2 gels. Through comparative analyses in the presence or absence of these factors, most studies conclude that increased levels of perfusion impart better clinical outcomes such as increased limb control. One exception was noted in a study by Soubeyrand et al., who showed that in a hemorrhagic insult, the use of vasodilating agents paradoxically increased the level of injury and reduced limb control [23].

Computational models are complementary tools to discern the effects of compression on spinal arterial blood flow and can provide insight into risk attributable to various types of injury. Moreover, parametric computational analyses can be performed to assess the effect of increasingly complex and severe compressions, and thus provide a basis for refining the thresholds for clinical intervention. Persson et. al. developed a finite-element model (FEM) of the spinal cord that included the cerebral spinal fluid (CSF) and dura mater to study the protective mechanical dampening of the tissue surrounding the spinal cord, which is exceedingly difficult to assess within an in vivo setting particularly when compression is applied from the anterior direction [30]. This study implemented a pellet thrust that caused acute deformation of the spinal cord, and concluded that the thickness of the CSF and dura mater are critical determinants of their protective capacity. Furthermore, they observed that upon impact the spinal cord begins to deform prior to subdural collapse, which confirms the presence of force transmission through the CSF. While this study did not assess the magnitude of neural damage, it did enhance understanding of spinal cord behavior under compression. Greaves et. al. use a three dimensional (3-D) FEM to study the relationship between various injury mechanisms and maximal strains within the spinal cord, with model validation demonstrated by comparison of predictions to published experimental data, specifically compressions in the anterior, posterior, and axial directions [31].

In this study, we examine how sustained sub-clinical compressive mechanical loading of the spinal cord impacts local vascular blood flow. A 3-D finite element fluid-structure interaction model of the spinal cord, dura mater, CSF, anterior spinal artery, and primary branches is used to investigate various compressive loading scenarios in the sub-clinical range. The effect of compressive loading is quantified via the precent reduction of blood flow through the anterior spincal artery and five of its branches as compared to the unloaded state.

## Methods

### Geometry

**Figure 1:**
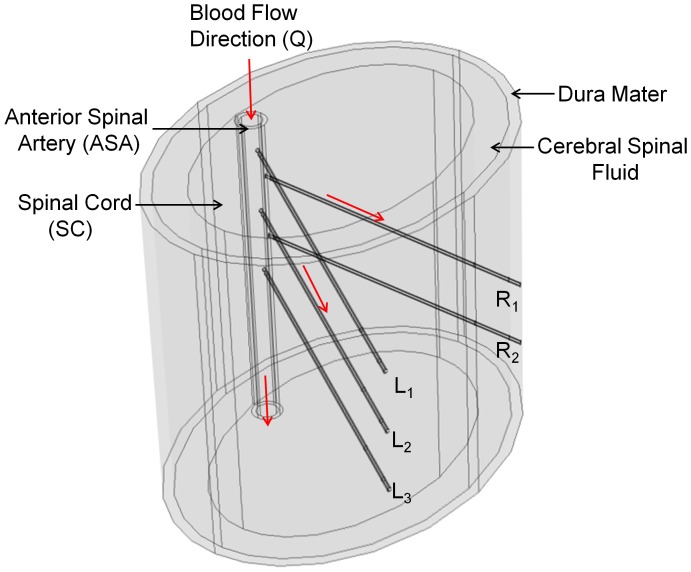
Schematic of simulation geometry comprising an ellipsoid segment of the cervical spinal cord surrounded by a cerebrospinal fluid (CSF) layer and dura mater. The anterior spinal artery (ASA) is within the spinal cord and has a luminal diameter of 1 mm and a thickness of 0.2 mm. Branches of the ASA are shown as L1, L2, L3, R1, and R2, all with luminal diameters of 0.14 mm and a thickness of 0.03 mm. Blood flow direction is indicated at the inlet of the ASA towards the ASA outlet and the 5 arterial branches, with a pressure gradient of 1 kPa and an average ASA inlet velocity of 0.3 m s^−1^.

A 3-D geometry of a longitudinal portion of the cervical spinal cord was developed using a commercial software package (COMSOL Multiphysics 4.0a, Comsol Inc.). The geometry consisted of a 1.5 cm segment of the anterior spinal artery (ASA), cervical spinal cord, cerebral spinal fluid (CSF) region, dura mater, and five arterial branches (L1, L2, L3, R1, and R2) that protrude into the spinal cord (Fig. 1). The thickness of the ASA wall, dura matter, CSF region and branch walls were 0.2 mm, 0.5 mm, 1.5 mm, and 0.03 mm, respectively [30]. The inner radii of the ASA and arterial branches were 0.5 mm and 0.07 mm, respectively [32].

By lumen volume, the ASA constitutes the majority of the spinal vasculature, and thus its occlusion is most likely to contribute to generalized spinal cord ischemia [29]. While posterior vasculature also plays a role in ischemia following injury, we sought to isolate the ischemic effects of compression caused by compromising the substantial blood supply mediated by the ASA. The specific selection of the number of vascular branches modeled (5) was based on the average of 4.6 sulcal arteries per cm in the spinal cord [33]. To avoid an overly complex geometry, microvascular branches were excluded from the modeling domain as it is expected that their response to compression would correspond to that of the primary branches.

### Governing equations and boundary conditions

A fluid-structure interaction (FSI) module was used to model the effect of spinal cord compression on blood flow through the spinal vasculature. Blood was modeled as a single-phased and incompressible Newtonian fluid. Blood flow through the lumen was assumed to be steady, laminar, and fully-developed, and was modeled using momentum and continuity equations as follows:
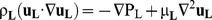
(1)




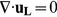
(2)Where 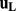, 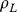 = 1060 kg m^−3^, 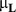 = 5×10^−3 ^Pa-s, and 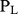 are respectively luminal blood velocity, density, viscosity, and pressure.

The structural mechanics of the ASA wall, spinal cord, CSF, and dura mater were described by the global stress equilibrium equations as follows:
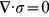
(3)where 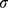 is the Cauchy stress tensor. ASA wall, spinal cord, CSF, and dura mater were modeled as linear elastic solids that exhibit mechanical isotropy and incompressibility (Table 1) [34]–[41].

**Table 1:** Material models and parameters used to simulate cervical spinal cord compression.

	Elastic Modulus (MPa)	Poisson’s ratio	Density (kg m^−3^)	Material model
Cervical spinal cord	1.4	0.40	1050	Linear elastic
Dura mater	80	0.49	1000	Linear elastic
Anterior spinal artery	1	0.45	1000	Linear elastic
Vascular branches	1	0.45	1000	Linear elastic
Cerebral spinal fluid	0.067	0.499	1040	Linear elastic

Solid domains were represented as linear elastic materials with constitutive mechanical descriptors extracted from the literature [34]–[41].

At the blood-ASA wall interface, the total force due to pressure and flow, 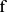, exerted on the ASA wall by the blood is given by:
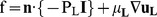
(4)where 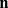 is the outward normal vector to the boundary and 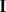 is the identity tensor.

A pressure gradient of 1 kPa was applied across the vessel inlet-outlets, yielding an average ASA inlet blood velocity of 0.3 m s^−1^. Zero displacement boundary conditions were assigned at the walls of vessel inlets and outlets to ensure that blood flow variations were not a consequence of cross-sectional area changes at the boundaries, but rather due to the presentation of applied loads and resultant deformations along the vessel lengths. Free displacement boundary conditions were applied in all other solid domain boundaries. To simulate the most clinically-relevant modes of compression, boundary loads were applied either anteriorly, posteriorly, or anteroposteriorly.

Stationary solutions to the described model were determined using COMSOL Multiphysics 4.0a. An adaptive physics-controlled meshing technique resulted in 291,621 tetrahedral elements for the model geometry. The mesh was developed with an average mesh quality of 0.614 and average element growth rate of 1.498, resulting in reasonable times for mesh generation as well as solution convergence. A relative tolerance of 10^−3^ was employed as a solution convergence criterion. A generalized minimum residual (GMRES) iterative method and geometric multigrid preconditioner comprised the linear system solver. A mesh-independent solution was defined when a less than 1% relative change in the maximum domain displacement and average inlet/outlet velocities occurred following additional mesh refinement.

Spinal compression was simulated with force vectors acting at the center of and normal to the outer surface of the dura mater, with applied forces ranging from 0–40 N. Dural occlusion is defined as the narrowing of the dura mater produced by a physical disruption, and here was calculated as the percent reduction in the short diameter of the ellipsoid composed of the spinal cord, CSF, and dura mater [42]. The range of compressive forces was selected to achieve cord deformations associated with sub-clinical dural occlusion (less than 10%) in all parametric studies. Vascular blood flow rates under compression were reported as the retained percentage of the corresponding vessel blood flow rate in the reference (unloaded) state. Using this model framework, we examine how compression magnitude and direction impact the perfusion capacity of the ASA and its primary branches.

## Results

**Figure 2:**
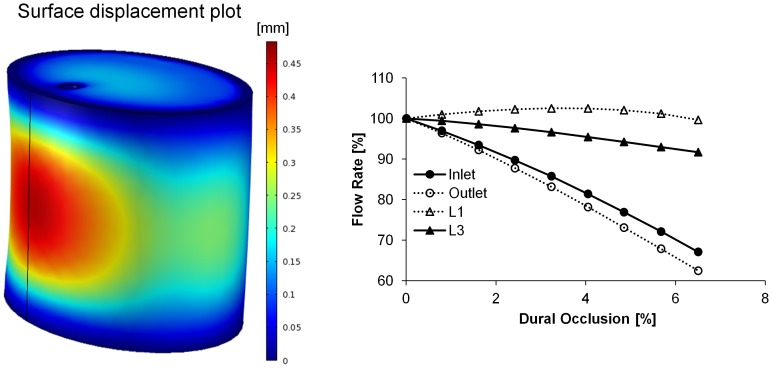
(Left) Representative surface plot of cervical spinal cord displacement under anterior loading. Colorimetric scale represents the induced displacement [mm]. (Right) Retained volumetric blood flow rate under anterior loading. The effect of anterior spinal compression on ASA and branch (L1 and L3) blood flow rates was assessed over the range of 0–6.5% dural occlusion, and the resultant change in arterial blood flow rates were calculated based on the percent of the baseline value (blood flow rate with no compressive loading).

**Figure 3:**
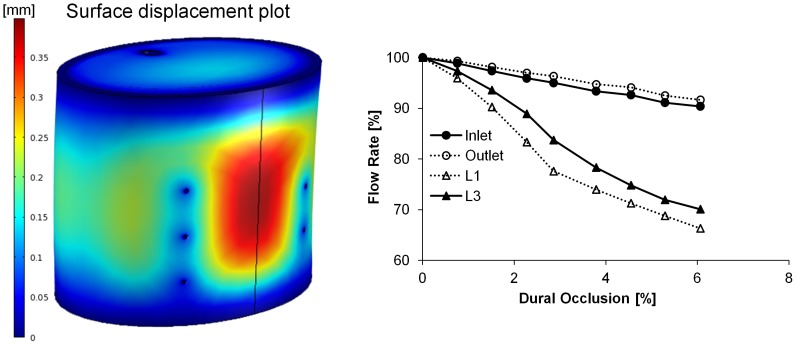
(Left) Representative surface plot of cervical spinal cord displacement under posterior loading. Colorimetric scale represents the induced displacement [mm]. (Right) Retained volumetric blood flow rate under posterior loading. The effect of posterior spinal compression on ASA and branch (L1 and L3) blood flow rates was assessed over the range of 0–6.05% dural occlusion, and the resultant change in arterial blood flow rates were calculated based on the percent of the baseline value (blood flow rate with no compressive loading).

**Figure 4:**
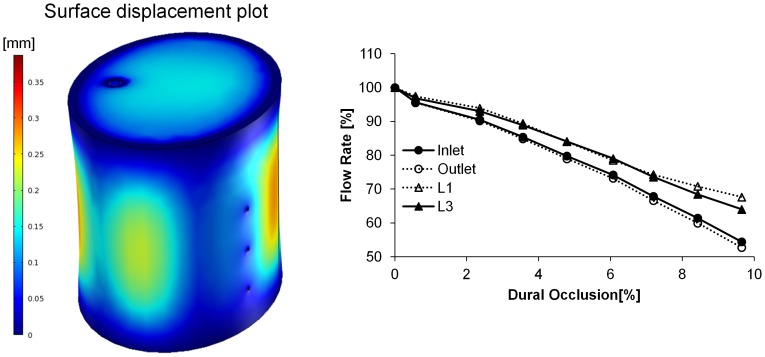
(Left) Representative surface plot of cervical spinal cord displacement under anteroposterior loading. Colorimetric scale represents the induced displacement [mm]. (Right) Retained volumetric blood flow rate under anteroposterior loading. The effect of anteroposterior spinal compression on ASA and branch (L1 and L3) blood flow rates was assessed over the range of 0–9.65% dural occlusion, and the resultant change in arterial blood flow rates were calculated based on the percent of the baseline value (blood flow rate with no compressive loading).

Post-processing of simulation results was performed to depict the ASA and select branch blood flow rates (L1 and L3) under various compressions, as the latter bracket the responses of the other branches (L2, R1, and R2) that have been omitted for clarity. Anterior loading differentially affected blood flow throughout the spinal vasculature, with notable reductions in the ASA inlet/outlet flow rates (Fig. 2). The ASA exhibited a monotonic and significant decrease in blood flow, with only 67% retention of baseline blood flow rate at 6.5% dural occlusion. Although minimal, a non-monotonic change in branch blood flow was observed as a consequence of an initial rise in the inlet-outlet pressure gradient, which was in turn a result of ASA deformation at the ASA-branch interface. Furthermore, branches nearest the inlet (L1 and R1) displayed higher flow rates before eventually declining, indicating a non-uniform response along the longitudinal spinal cord axis. The effect of posterior loading was qualitatively reversed, with branch blood flow significantly compromised and the ASA blood flow relatively insensitive to the induced dural occlusion (Fig. 3). Anteroposterior compression, simulated via application of simultaneous and equivalent loads to opposed surfaces of the dura mater, compromised blood flow through both the ASA and its branches (Fig. 4). The percent reduction of blood flow rate due to anteroposterior compression was most severe in the ASA, with an approximate 50% loss at 9.65% dural occlusion.

**Figure 5:**
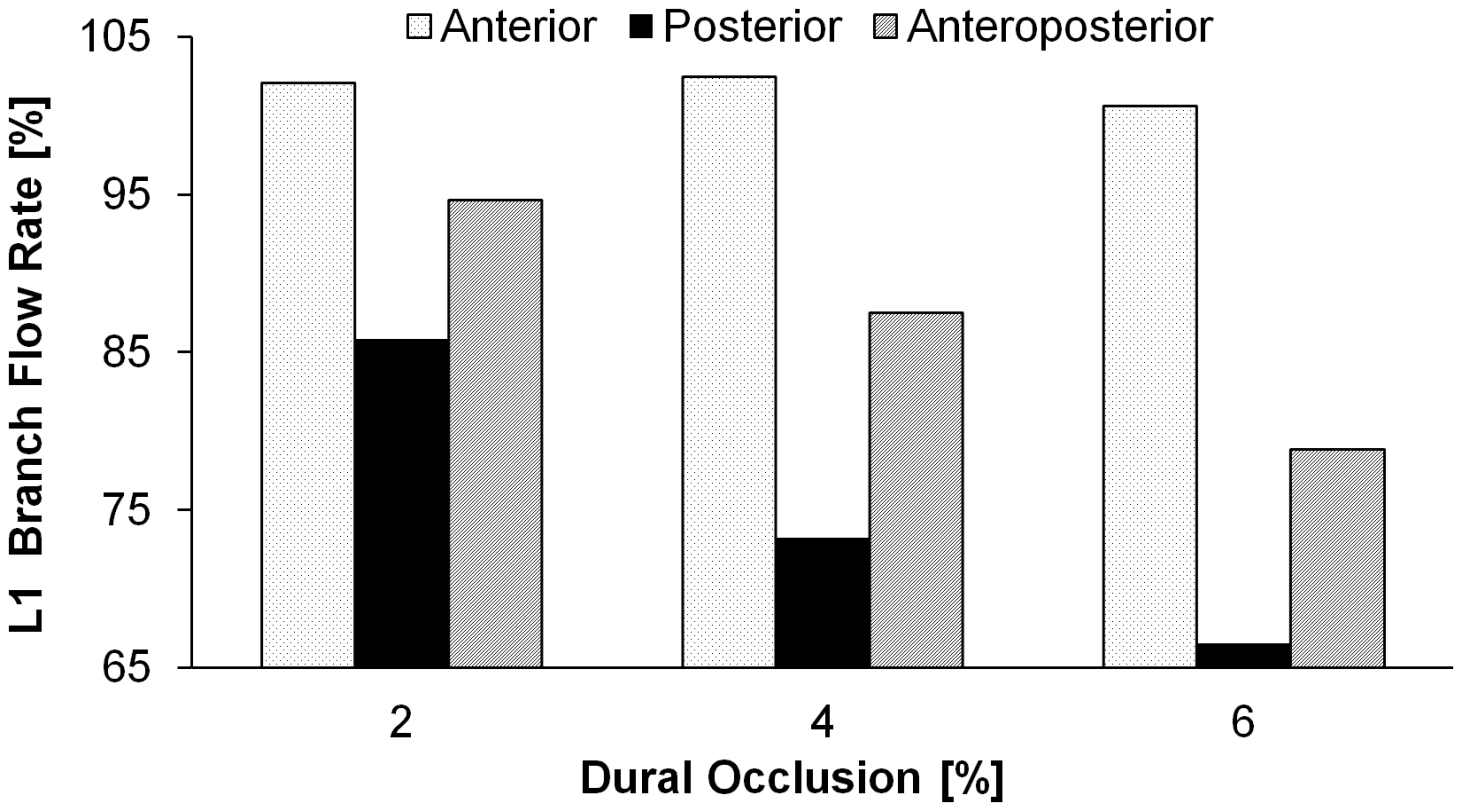
Retained L1 branch volumetric blood flow rate under various modes of spinal compression. Under equivalent degrees of dural occlusion, the induced reduction of branch blood flow rate varies with the orientation of compression.

Comparison between blood flow rates following various modes of compression indicates that the percent dural occlusion alone is an insufficient predictor of the ischemic potential following SCI (Fig. 5). At equivalent levels of dural occlusion, the predicted loss of branch (L1) blood flow was greater for purely posterior compressions as compared to the other examined modalities. Thus, these findings suggest that the decision to intervene following SCI should consider both the extent of occlusion and the orientation of cord compression.

## Discussion

The developed computational model of the cervical spinal cord provides insight on how compressive loading can compromise spinal vasculature function and informs speculation on the induced ischemic potential under various loading scenarios. Our findings suggest that the mode of compression should be considered in risk assessment and that certain modes potentially require intervention at traditionally sub-clinical levels.

Anterior loading resulted in a substantial reduction in ASA flow, which is understood as a consequence of the vessel proximity to the loading surface and the resultant vessel deformation. Under this mode of loading, the luminal deformation of the arterial branches is minimal and their flow rates are essentially unchanged. However, we speculate that the change in local ASA wall mechanics could evoke an adaptive remodeling response, which may reduce the inherent ability of this vessel to compensate for transient changes in blood pressure or flow. Thus, although arterial branch flow and presumably the perfusion of local tissue are not immediately affected, aberrant ASA mechanics give rise to a potential for chronic complications following anterior compression.

Posterior compression minimally affected ASA blood flow but significantly altered branch flows. Here again, the change in blood flow can be understood a consequence of vessel distance from the loading surface of the dura mater. As compared to anterior compression, the potential for an acute ischemic event in this scenario is elevated due to the dramatic reduction of microvascular flow. Our findings suggest the need to define a lower threshold for intervention in posterior cervical spinal cord injuries, both in terms of compression time and severity.

At equivalent degrees of dural occlusion, the loss of branch blood flow under anteroposterior compression was intermediate to predictions for purely posterior or anterior loading. Moreover, this compression scenario displayed a monotonic and significant reduction of both ASA and branch blood flow rates with increasing dural occlusion. Taken together, findings suggest that anteroposterior compression substantially limits the vascular capacity for general spinal cord perfusion by virtue of compromising flow throughout the vascular bed.

There have been a number of pre-clinical studies that indirectly support and can be reconsidered in light of our presented findings. Ramsey et.al. carried out a series of experiments in monkeys following the creation of a controlled epidural compression via balloons introduced percutaneously both anterior and posterior to the spinal cord [43], [44]. Selective spinal angiography was performed to assess in vivo perfusion. While a severe compression, both anterior and posterior, resulted in significant decline in spinal cord perfusion, it was noted that a moderate anterior compression caused microvascular deformation and therefore a decline in perfusion within the gray matter without a significant change in ASA diameter. Moderate posterior compressions were however not performed. In similar studies, Vlajic et.al. reported a decline in spinal cord perfusion after spinal cord compression in rabbits, and found that vessels in gray matter were less often filled than the peripheral vasculature, such as the ASA. Moreover, the study concluded that vessels studied after decompression exhibited recovered filling, which indicates a reversible mechanically-mediated phenomenon [45].

The difference in perfusion deficit as predicted by this model for anterior versus posterior compression may underlie disproportionate neurological deficit observed in certain posterior spinal compression syndromes. Posterior location of epidural abscess presents more frequently with paraplegia or quadriplegia than anterior epidural abscesses (30.6% versus 7.3%) [46]. Our findings support the early decompression of compressive epidural abscesses presenting with neurological deficits, especially when on the posterior side of the spinal cord. We also speculate the need for a lower threshold for intervention in posteriorly located lesions since a greater degree of vascular compromise might predispose these patients to significant neurological deficits. Traumatic perched or jumped facets commonly present posterior compression in clinical practice. Similar to the posterior epidural abscesses, the posterior compression associated with perched facets is associated with disproportionately higher incidence of neurological deficits from cord compression [47], [48]. More importantly, emergent decompression within four hours results in significant recovery of neurological deficits, possibly suggesting an underlying reversible ischemia [47]. Moreover, a delay in time to decompression (mean delay time of 25 hours) results in significantly worse recovery in a facet dislocation group as compared to a non-facet dislocation group [48].

### Study Limitations

The limitations of our study must be considered for proper interpretation of the presented findings. Firstly, the computational model and thus resulting predictions have not been validated in vivo, although qualitatively there is general agreement with reported pre-clinical and clinical observations [43]–[46]. Moreover, at approximately 0.5 mm cord displacement, Greaves et al. conclude a 10% canal stenosis had occurred [31], which is comaprtively elevated but close to the values predicted by our model for similar degrees of injury. The isotropic linear elastic material models used for all solid domains do not reflect the established mechanical behavior of these tissues, but were adapted in light of the near equivalency to nonlinear anisotropic elastic behavior under small deformations (<10% strain in all simulated scenarios) and the notable increase in solution speed compared to increasingly complex material models. Additionally, the CSF was considered as an incompressible linear elastic solid, with fluid-like properties and a low-shear modulus, as done in previous studies of brain and spinal cord with the similar intent to alleviate computational burden [49]. Blood was modeled as a Newtonian fluid with non-pulsatile flow, both of which have been shown to minimally impact computational solutions involving vascular fluid mechanics. Finally, there is a limitation in the depicted collateral circulation of the ASA, which has more than one inlet and thus more potential for compensatory flow following compression.

## Conclusion

Computational modeling was used to examine how mild compression of the cervical spinal cord impacts local blood flow in various states of spinal cord health. Our findings suggest that SCI causing spinal dural occlusion well-below the documented clinical threshold of 35% can compromise local blood flow, with the most dramatic effects on branch flows induced by posterior compression. Computational modeling provides insight to a discrepancy noted in clinical practice for different directions of spinal compression and suggests that alterations in local blood flow is a potential contributor to inconsistence patient outcomes.
